# Deltoid Intramuscular Injections: A Systematic Review of Underlying Neurovascular Structures to the Muscle and Proposing a Relatively Safer Site

**DOI:** 10.7759/cureus.24172

**Published:** 2022-04-15

**Authors:** Sundip Charmode, Shelja Sharma, Sudhir Shyam Kushwaha, Simmi Mehra, Sarah S Sangma, Vivek Mishra

**Affiliations:** 1 Anatomy, All India Institute of Medical Sciences, Rajkot, IND; 2 Anatomy, All India Institute of Medical Sciences, Gorakhpur, IND; 3 Department of Orthopedics, All India Institute of Medical Sciences, Gorakhpur, IND

**Keywords:** needle depth, axillary nerve, safe site for injection, deltoid muscle, intramuscular injections

## Abstract

The deltoid is the preferred site for intramuscular injection (IMI) because of its easy accessibility for drug and vaccine administration. Government immunization advisories, standard anatomy textbooks, and researchers have proposed various injection techniques and sites, but specific guidelines are lacking for the administration of IMIs in the increasingly used deltoid site. This study analyzes the procedures of administering IMIs in the deltoid related to the neurovascular network underlying the muscle and proposes a preferred site with the least chance of injury. The review protocol was submitted with PROSPERO (ID: 319251). PubMed, Google Scholar, and Websites of National Public Health Agencies were searched from 1950 up to 2022 for articles, advisories, and National Immunization Guidelines using Medical Subject Headings (MeSH) terms, including IMIs, deltoid muscle, safe injection sites, to identify recommendations for safer sites and techniques of administering deltoid IMIs. All the authors strictly adhered to a well-developed registered review protocol throughout the study and followed the risk of bias in systematic reviews (ROBIS) guidance tool. The proposed sites and landmark data were tabulated, and each site was analyzed based on the underlying neurovascular structures. Data were depicted by self-generated images.

The initial search identified 174 articles. After applying the inclusion and exclusion criteria, 57 articles were shortlisted. Out of the 39 selected articles, 18 focused on the administration of deltoid IMIs, whereas seven focused on the variations in the underlying neurovascular structures in proximity to the deltoid muscle. The remaining 14 articles were the immunization guides issued by the National Public Health Agencies of the Government of India and abroad, whose data was used for comparison. Twelve deltoid IMI sites and techniques were identified. A site 1-3 fingerbreadths/5 cm below the mid-acromion point (7 studies); mid-deltoid site/densest part of the deltoid (1 study); a site at the middle third of the deltoid muscle (1 study); triangular injection site (1 study). Limitations included the unavailability of free access to complete text in many articles resulting in exclusion. The area around the shoulder joint and up to the lower level of the intertubercular sulcus is highly vascularized by the presence of many anomalous arterial patterns. To avoid injury, a safer site is proposed of 5 fingerbreadths/10 cm below the midpoint of the lateral border of the acromion. The authors received no specific funding for this study except for the journal publication charges.

## Introduction and background

**Figure 1:**
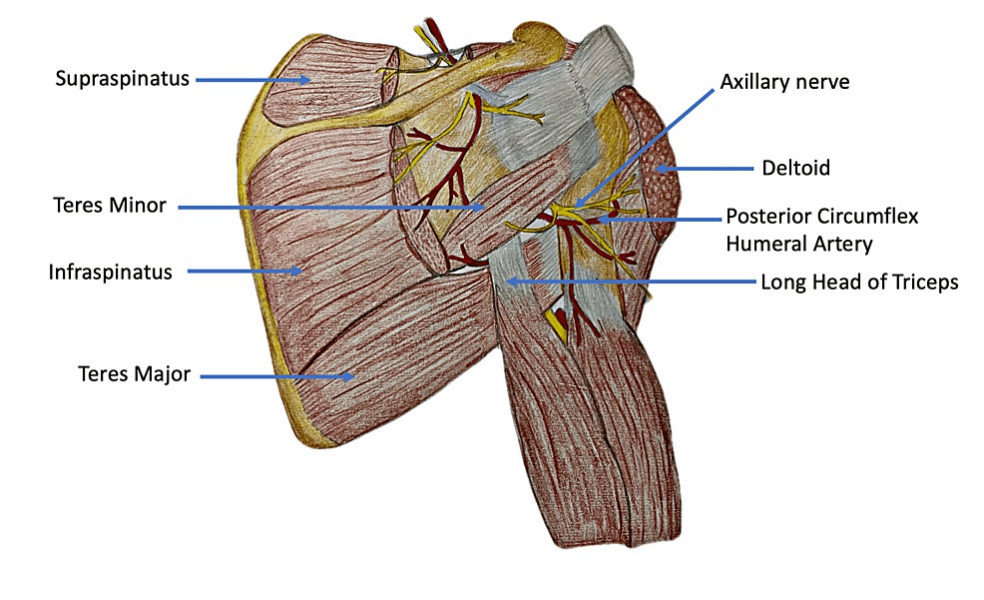
Structures underlying the deltoid muscle. The image was created and edited by Dr. Shalom Philip, Senior Resident, AIIMS Rajkot

Intramuscular injections (IMIs) are among the most common medical procedures performed in any healthcare center [1]. Globally, the deltoid is the preferred IMI site in clinical practice [1]. Many other IMI sites have been considered over the deltoid based on the risk of injury to the underlying vessels and nerves. However, a paucity of uniform guidelines and algorithms persists for IMI administration by healthcare professionals [2]. This study analyzes the procedures of IMI administration in the deltoid in relation to the arterial network underlying the muscle. Our study proposes a site preferred to the deltoid for IMIs with the least chance of injury to neurovascular structures. Figure 1 and Table 1 present the structures underlying the deltoid muscle.

**Table 1:** Structures underlying the deltoid muscle [3]

Sr. no	Underlying Muscle and Tendon Structures	Underlying Neurovascular Structures
1	Four rotator cuff muscles	Axillary nerve (AXN)
2	Pectoralis major	Anterior circumflex humeral artery (ACHA)
3	Tendon of pectoralis minor	Posterior circumflex humeral artery (PCHA)
4	Tendons of coracobrachialis	
5	Long and short heads of biceps brachii	
6	Long and lateral heads of the triceps brachii	

## Review

Methods

To reduce the risk of bias in the study, a systematic review protocol was prepared and submitted with PROSPERO at the Centre for Reviews and Dissemination, University of York (ID: 319251). The review protocol can be accessed from https://www.crd.york.ac.uk/prospero/.

Eligibility criteria

The articles which satisfied the inclusion and exclusion criteria were eligible for review. Inclusion criteria were complete articles published between January 1, 1950, and January 31, 2022, authorship by both foreign and Indian authors, immunization guidelines and advisories issued by national public health agencies, and all articles related to deltoid IMIs. Excluded articles were published before January 1, 1950, and after January 31, 2022, those focusing on topics other than deltoid IMIs, and those accessible through abstracts only. There was a restriction for the non-English language of publications.

Search strategy

A scoping review was conducted of articles published from 1950 to 2022 on PubMed, Google Scholar, and National Immunization Guidelines using these Medical Subject Headings (MeSH) terms: IMIs, deltoid muscle, safe site for injection, axillary nerve, needle depth, nursing practice, post-injection complications, posterior circumflex humeral artery, and anterior circumflex humeral artery. The citation search was carried out for all the selected articles in the study.

Study selection

After applying inclusion and exclusion criteria, two authors (SC and SS) independently assessed all of the titles, abstracts, articles, and guidelines found during the initial search, and relevant publications were shortlisted. All of the shortlisted full publications were downloaded and independently examined for relevant data using the data extraction checklist prepared by both authors. The authors and the methods of the investigations were not hidden from the reviewers. Any differences were settled through conversation or the involvement of a third reviewer (VM). The special data needed for the review is mentioned on the checklist (Table 2). We chose and incorporated the articles that contained this information.

**Table 2:** Checklist for data extraction from the selected articles. IMI: Intramuscular injection

Sr. no	Data Collected	Data Ignored
1	Whether any new site for deltoid IMI is proposed	Information about any technique of administering deltoid IMI
2	Whether any reason is mentioned for recommending the proposed deltoid IM site	Information about importance of needle depth and angle of needle insertion at the time of administering IM
3	Whether any site is not recommended or marked as high risk for deltoid IMI	Information about importance of measuring the thickness of subcutaneous tissue at the IM site
4	Whether any reasons are mentioned for not recommending any deltoid IM site	Information about importance of choosing the type of needle while administering IMI
5	Whether any neurovascular structures are underlying the not-proposed IM site	
6	Mention of any post-injection complications occurring after deltoid IMI	
7	Critical appraisal of currently used techniques for deltoid IMI	
8	Reference to common mistakes committed by healthcare workers in administering deltoid IMI	
9	Variations in the pattern of neurovascular structures in proximity to the deltoid muscle	

Data extraction process

The authors created a data extraction form that they used to collect data from any two papers they chose, and it was verified after the pilot trial. Two reviewers (SC and SS) worked separately to gather data from all of the studies that were included. Disagreements were addressed through dialogue and the participation of a third independent reviewer (VM). The following features of the study were gathered: (i) the research author; (ii) the study design; (iii) the country of publication; (iv) the number of participants; (v) the participants’ age group; (vi) the participants’ gender; and (vii) the participants’ ethnicity. The following information was gathered about the IMI location in the research and control groups: any new suggested deltoid IMI location; (ii) the distance between the recommended IMI site and the mid-acromial point; (iii) any underlying neurovascular structures to the recommended deltoid IMI site; (iv) any post-injection complications occurring after the deltoid IMI at the recommended site; (vi) any site not recommended or marked as high risk for deltoid IMI; (vii) any underlying neurovascular structures to the not-recommended deltoid IMI site; and (vii) any underlying neurovascular structures to the not-recommended deltoid IMI.

Risk of bias

Two reviewers (SC and SS) independently conducted the risk of bias assessment that was included in the data extraction form. The risk of bias in systematic reviews (ROBIS) tool was used to assess the risk of bias in our review study [4].

Results

Literature Search

**Figure 2:**
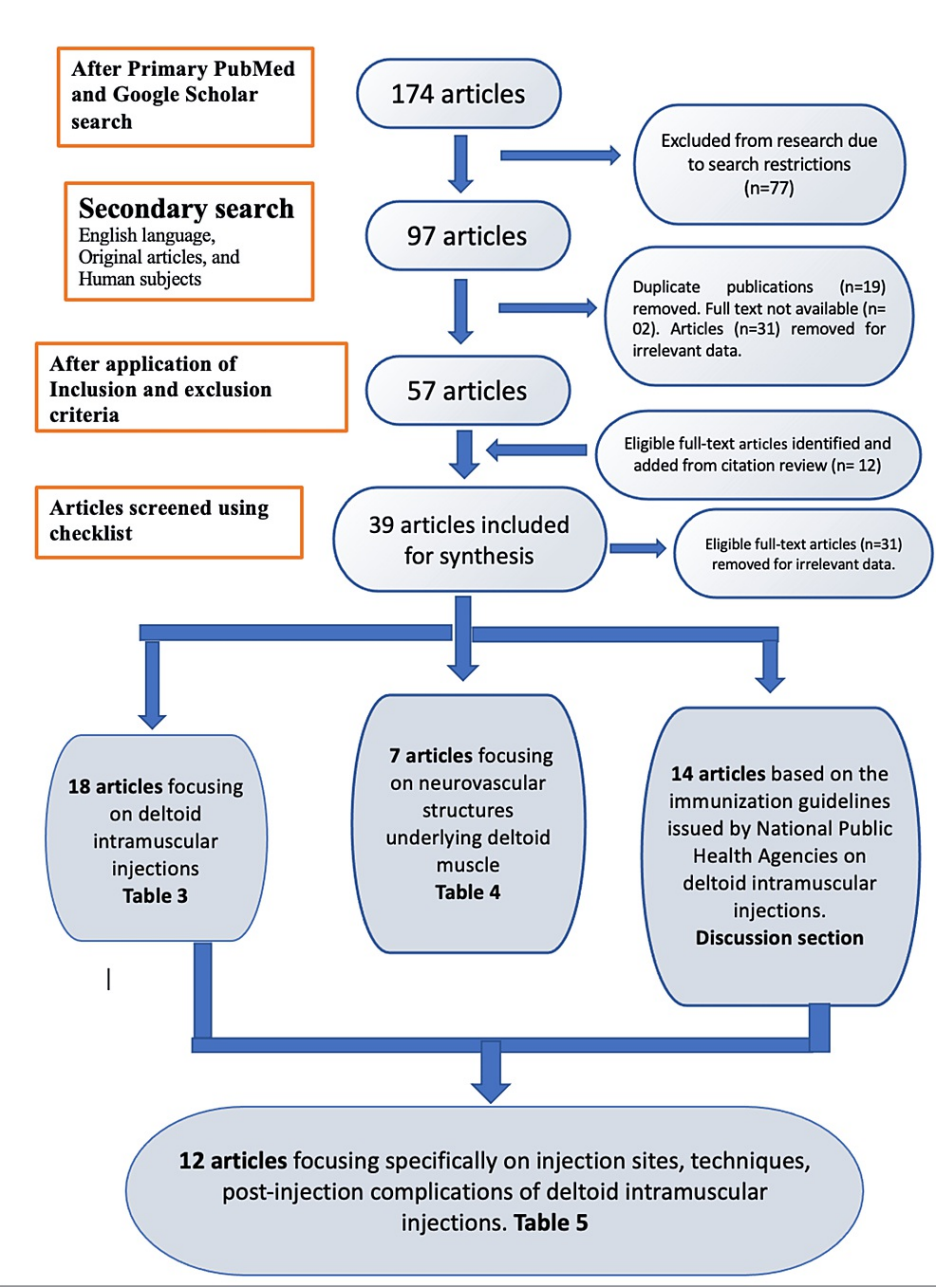
PRISMA flowchart of the systematic literature search performed for articles focused on administering deltoid intramuscular injections.

There were 174 published papers found after the initial search. After filtering for the English language, original content, and human involvement, 97 articles remained. Duplicate articles (n=19) were deleted, and two reviewers independently assessed 97 publications. The same two independent reviewers independently examined 12 papers found through citation searches for eligibility against the pre-specified inclusion criteria. Disagreements were settled by conversation. After applying the inclusion and exclusion criteria, 57 articles remained. Thirty-one publications were excluded due to irrelevant text and two publications due to unavailability of the full text. Based on each author’s appraisal and cross-verification, 39 papers were selected for synthesis. Eighteen papers were eligible for inclusion and exclusion based on the inclusion and exclusion criteria but were eliminated after reviewers screened them for irrelevant data that was outside the scope of the current review. They are namely Wempe 1961 [5], Taylor 2021 [6], and Micallef et al. 2020 [7]. Figure 2 shows the Preferred Reporting Items for Systematic Reviews and Meta-Analyses (PRISMA) flowchart, which shows the step-by-step literature search and consideration/rejection procedure.

**Table 3:** Characteristics of 18 articles focusing on deltoid intramuscular injection. AXN: axillary nerve; IMI: intramuscular injection

Sr. no.	Title of the article	Authors and publication year & country of publication	Study design	Number of participants & gender	Participants age group	Ethnicity of participants	Observations/Recommendations
1	The right site for IM injections	Winslow, 1996 [8]; USA	Survey	Not available	Not available	American Indian population	Of IMI sites (deltoid, vastus lateralis, dorsogluteal, ventrogluteal), only the ventrogluteal site was not associated with any adverse effects
2	Appropriate site for intramuscular injection in the deltoid muscle evaluated in 35 cadaverous arms	Nakatani et al., 2000 [9]; Japan	Cadaveric study	35	Not available	Asian population	AXN is frequently positioned 5 cm below the midpoint of lateral border of acromion; therefore, this site is unsuitable for IMI due to risk of injury to this nerve
3	The deltoid intramuscular injection site in the adult. Current practice among general practitioners and practice nurses	McGarvey and Hooper, 2005 [10]; Ireland	Public survey	Not available	Adults	White population	Injury to structures underneath the deltoid muscle can be avoided using appropriate needle lengths. Current IMI techniques at the deltoid site are deficient in many respects. Both general practitioners and practice nurses have a poor understanding of structures are at risk from IMI in the deltoid region
4	The problem of using deltoid muscle for intramuscular injection	Fujimoto, 2007 [11]; Japan	Cadaveric study	14	Not available	Asian population	The deltoid muscle is not necessarily safe or appropriate for IMI due to the possibility of AXN injury; instead, the ventrogluteal site is the first choice
5	Iatrogenic axillary neuropathy after intramuscular injection of the deltoid muscle	Davidson et al., 2007 [12]; USA	Case report	Male	26 years old	American Indian population	Deltoid IMI may result in direct mechanical trauma to the anterior branch of the AXN resulting in axillary mononeuropathy with axonal loss
6	Deltoid contracture: a case with multiple muscle contractures	Chen et al., 2008 [13]; Taiwan	Case report	Not available	Not available	Asian population	The case-patient experienced muscular contracture induced by needle injection, regardless of age, medication, and injection site
7	An evidence-based protocol for the prevention of upper arm injury related to vaccine administration (UAIRVA)	Cook, 2011 [14]; Australia	Cadaveric study	Not available	Adults > 65 years	White population	The midpoint of the deltoid muscle, defined as the middle point between the acromion and the deltoid tuberosity with the arm abducted to 60°, is a safe site for IMI
8	Post-vaccination frozen shoulder syndrome. Report of three cases	Degreef and Debeer, 2012 [15]; Belgium	Case report	Three	Not available	White population	Frozen shoulder syndrome can be a severe manifestation of vaccination-related shoulder dysfunction
9	Teaching best evidence: Deltoid intramuscular injection technique	Davidson and Rourke, 2013 [16]; Canada	Case report	Three	Not available	White population	The “axillary triangle method” was proposed. Three modifications should be urgently implemented in nursing training programs: Nursing students must be taught about structures at risk with IMIs. Nursing students should measure their own fingers to decide a 4-cm range to use for landmarking the deltoid site. Nursing students must be educated to choose needle length based on the client’s body weight
10	Influence of skin-to-muscle and muscle-to-bone thickness on depth of needle penetration in adults at the deltoid intramuscular injection site	Shankar et al., 2014 [17]; India	Analytical cross-sectional study	200 (100 male and 100 female)	Adult age group	Asian population	Over-penetration of deltoid IMI is more prevalent compared with under-penetration; thus, modification of technique of IMI is recommended based on the individual patient’s body type
11	Best vaccination practice and medically attended injection site events following deltoid intramuscular injection	Cook, 2015 [18]; Australia	Review study	Not available	Not available	White population	Best practice recommendations are proposed: Selection of a “safe” site for injection. Individualizing needle length for muscle penetration. Using a standardized injection technique and skin preparation before injection
12	Risk of bursitis and other injuries and dysfunctions of the shoulder following vaccinations	Martín et al., 2017 [19]; Spain	Review study	Not available	Adults	White population	Subdeltoid or subacromial bursitis and other shoulder lesions are more likely to result from a poor injection technique, including site, angle, needle size, and failure to consider patient’s characteristic (i.e., sex, body weight, and physical constitution)
13	Establishing a new appropriate intramuscular injection site in the deltoid muscle	Nakajima et al., 2017 [1]; Japan	Prospective study	30 (15 male, 15 female)	Age > 17 years	Asian population	A perpendicular/vertical line extending from the midpoint of lateral border of acromion and intersecting with another line that connects the upper ends of the anterior axillary line and the posterior axillary line is the intersection point proposed as the safe site for IMI
14	Upper limb nerve injuries caused by intramuscular injection or routine venipuncture	Kim et al., 2017 [20]; Korea	Review study	Not available	Not available	Asian population	The recommended injection site is the midpoint of the deltoid muscle (the densest part of the muscle) or approximately 3–5 cm below the lower edge of the acromion midway between acromion and deltoid tuberosity
15	Efficacy and safety in intramuscular injection techniques using ultrasonographic data	Tanioka et al. 2018 [21]; Japan	USG-based study	136	Not available	Asian population	Use of a 23-G 25-mm injection needle is proposed in the case of a deltoid IMI site, in the absence of notable obesity
16	Shoulder injury related to vaccine administration and other injection site events	Bancsi et al., 2019 [22]; USA	Review study	Not available	Not available	White population	The proposed general guidelines to identify the upper border of the injection site by placing two or three fingers across the deltoid muscle below the acromion process
17	Intramuscular injections	Polania Gutierrez and Munakomi, 2021 [23]	Book chapter	Not applicable	Not applicable	Not applicable	Use these selection techniques for deltoid IMI: Site: 2.5–5 cm below the acromion process; needle length: 16–32 mm (children), 25–38 mm (adults); Drug volume: 2 mL or less
18	Statistical estimation of deltoid subcutaneous fat pad thickness: implications for needle length for vaccination	Sebro 2022 [24]; USA	Retrospective cohort study	386	The age range was 19 to 93	White population	Per the current Centers for Disease Control and Prevention guidelines, deltoid IMI may result in subcutaneous injection and thereby reducing the vaccine efficacy in females and overweight persons

**Table 4:** Characteristics of seven articles focusing on variations in the pattern of neurovascular structures in proximity to the deltoid muscle. ACHA: anterior circumflex humeral artery; PCHA: posterior circumflex humeral artery

Sr. no.	Title of the Article	Author and publication year & country of publication	Study design	Number of participants & gender	Participants age group	Ethnicity of participants	Observations/suggestions/recommendations
1	Anatomical variations of the deltoid artery	Bunker et al., 2013 [25]; UK	Prospective observational study	100 deltopectoral approaches were studied	Aged > 18 years	White population	The thoracoacromial artery provides two collaterals in relation to the ventral surface of the deltoid muscle. The first, termed the deltoid artery, runs in front of the deltoid muscle, near the deltopectoral line. In 53% of cases, this deltoid artery forms the first upper collateral branch, running 3 cm below the collarbone. The second, termed the acromial artery, runs deep to the anterior part of deltoid muscle, near the clavicle. The deltoid branch of thoracoacromial artery accompanies the cephalic vein in deltopectoral groove and supplies the deltoid
2	The vascular territory of the acromio-thoracic axis	Reid and Taylor, 1984 [26]; Australia	Cadaveric study	110 cadaver dissections	Not available	White population	Two common variants of the deltoid artery were found. In type I (71%), it passes through interval and tunnels in the deltoid muscle without hitting the cephalic vein. However, in type II (21%), it intersects the hole, reaches the cephalic vein, and then runs down, medially to and behind it, creating many small arterial branches that return through the opening in pectoralis major. Several small variances were also observed (8%). The deltoid artery supplies the skin over the shoulder by numerous small branches that emerge from the intramuscular septa of the deltoid muscle. In addition, a large axial artery was noted. In most cases, this artery arose from the deltoid artery or its acromial branch and coursed laterally
3	Anatomy of the terminal branch of the PCHA: relevance to the deltopectoral approach to the shoulder	Smith et al., 2016 [27]; UK	Observational study	100 deltopectoral approaches were studied	Age > 18 years	White population	In a study of 92 patients, for all participants, the terminal branch of PCHA crossed the space between the deltoid and the proximal humerus and was prone to injury during insertion of the blade of a retractor during the deltopectoral approach to the shoulder. Of the 92 patients, 75 (75%) had a single vessel, 16 (16%) had a double vessel, and one had a triple vessel
4	Smith et al., 2016 [27]; Gilbert and Nelson, 2022 [28]						The PCHA arises at the distal border of the subscapularis and runs backward with AXN through quadrangular space to run in relation to the surgical neck of humerus. The PCHA has a descending branch that anastomoses with the deltoid branch of the profunda brachii artery and with the ACHA and acromial branches of the suprascapular and thoracoacromial artery. The PCHA supplies dorsal and central parts of deltoid muscle
5	Anatomy, shoulder and upper limb, anterior humeral circumflex artery	Gilbert and Nelson, 2022 [28]; USA	Book chapter	Not applicable	Not applicable	Not applicable	The ACHA supplies the anterior part of the deltoid muscle in 63% of cases [26]. The ACHA forms an anastomosis with the PCHA and other arterial branches, such as the profunda brachii and the acromio-thoracic artery. Thus, the ACHA supplies the anterior part of deltoid muscles, head of humerus, glenohumeral joint, and Teres major and minor
6	Gray’s anatomy: the anatomical basis of clinical anatomy, 42^nd^ ed	Stranding, 2020 [29]; UK	Book chapter	Not applicable	Not applicable	Not applicable	In about 33% of cases, the subscapular artery can arise from a common trunk with PCHA. Occasionally the subscapular, circumflex humeral, and profunda brachii arteries arise from a common trunk. In some cases, the PCHA may arise from profunda brachii artery. Therefore, branching and distribution patterns of different branches and their networks are quite variable around the shoulder joint and proximal humerus
7	Determination of deltoid fat pad thickness. Implications for needle length in adult immunization	Poland et al., 1997 [30]; USA	Prospective study	220 healthy health care workers (126 women, 94 men)	Adult age group	American Indian population	A deltoid IMI is defined as an injection with penetration of the muscle by 5 mm or more, with 2 mm of needle superficial to the skin to aid in needle retrieval in the event of an accidental needle break
8	General recommendations on immunization: recommendations of the Advisory Committee on Immunization Practices	Centers for Disease Control and Prevention (CDC), 2011 [31]	Immunization report	Not applicable	Not applicable	Not applicable	When considering IMI sites, a clinician should prefer a site that is at a safe distance from nerves, large blood vessels, and bones, free from injury, abscesses, tenderness, necrosis, abrasions, and other pathologies, and sufficiently large to accommodate the volume of medication to be administered. The deltoid site is preferred because it is easily accessible for clinicians and for patients to expose

**Table 5:** Twelve articles recommending sites for deltoid intramuscular injections. AXN: axillary nerve; IMI: intramuscular injection

Sr. no.	Authors and publication year & country of publication	Study design	Number of participants & gender	Participants age group	Ethnicity of participants	Proposed site/technique
1	Davidson et al., 2007 [12]; USA	Case report	Male	26 years old	American Indian population	Site is 1–3 fingerbreadths (5 cm) below the mid-acromion, and it is frequently used in clinical settings in Japan. The site is shown in a self-generated image in Figure 3
	Beyea and Nicoll, 1995 [32]; UK	Integrative review	Literature of last seven decades	Not applicable	White population	
2	Kozieret al., 2010 [33]; Australia	Nursing manual	Not applicable	Not applicable	White population	The student nurse should use four fingers, placing the little finger on the acromion process, and three fingers below
3	Treas and Wilkinson, 2014 [34]; USA	Based on multi-cultural, multi-generational, Asian-American family case studies			Asian-American population	A triangular injection site is proposed. The apex is directed at a point of intersection between the line connecting the upper ends of the anterior and posterior axillary lines and a vertical line extending from the mid-acromion point. The base is formed by a horizontal line positioned 1–3 fingerbreadths (5 cm) below the acromion. The site is shown in a self-generated image in Figure 4
	Gray et al., 2009 [35]; UK	Pragmatic Review	Not applicable	Not applicable	White population	
	Rodger and King, 2000 [36]; UK	Literature review	Not applicable	Not applicable	White population	
4	Funnell et al., 2005 [37]; Australia	Nursing manual	Not applicable	Not applicable	Australian–New Zealand white population	Site is at the middle third of the deltoid muscle, with acromion as the origin of the deltoid and the deltoid tuberosity as the insertion of the deltoid muscle. This site is the densest part of deltoid. The site is shown in a self-generated image in Figure 5
5	Kim et al., 2017 [20]; Korea	Review study	Not applicable	Not applicable	Asian population	A mid-deltoid site is proposed, with the acromion as the origin of the deltoid muscle and the deltoid tuberosity as the insertion of the deltoid muscle. The site is shown in a self-generated image in Figure 6
6	Cocoman and Murray, 2008 [38]; Ireland	Review study	Not applicable	Not applicable	White population	An injection site is recommended approximately 3–5 cm below the lower edge of the acromion, but this site is also unsafe due to the presence of the AXN
7	Nakajima et al., 2017 [1]; Japan	Prospective study	30 (15 male, 15 female)	Age > 17 years	Asian population	A new site is proposed: divide the superolateral margin of acromion into three points: posterior (a); mid-portion (b); and anterior (c). Draw a line between the upper corners of anterior and posterior fold line (line AB). Finally, draw a perpendicular line from both points (a) and (b) of acromion to AB line. The zone between the halfway point of the a-AB line and the lower one-third of the b-AB line may be safe for IMIs. The site is shown in a self-generated image in Figure 7
8	Cook, 2011 [14]; Australia	Cadaveric study	Not available	Adults > 65 years	White population	A safer IMI site is recommended that is 7.4 cm below the mid-acromion in both sexes due to the course of the AXN and position of the subacromial/subdeltoid bursa. The site is shown in a self-generated image in Figure 8
9	Lammon et al., 1995 [39]; UK	Nursing manual	Not applicable	Not applicable	White population	Many nursing textbooks illustrate no AXN but do show a radial nerve, and state: “You must inject the medication into the densest part of deltoid to avoid the radial nerve and artery”

**Figure 3:**
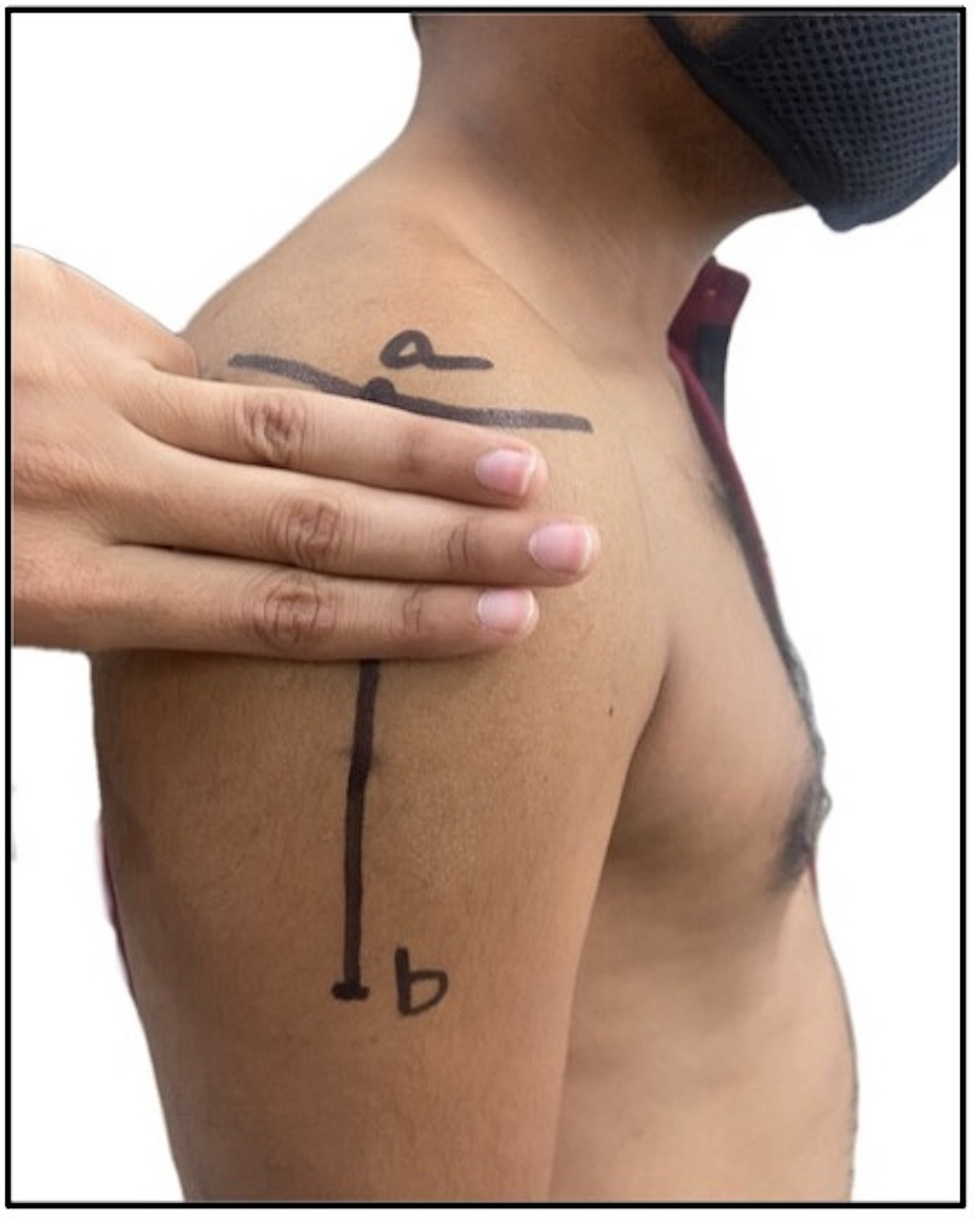
Site 3 fingerbreadths (5 cm) below the mid-acromion: a) midpoint of lateral border of acromion; b) deltoid tuberosity.

**Figure 4:**
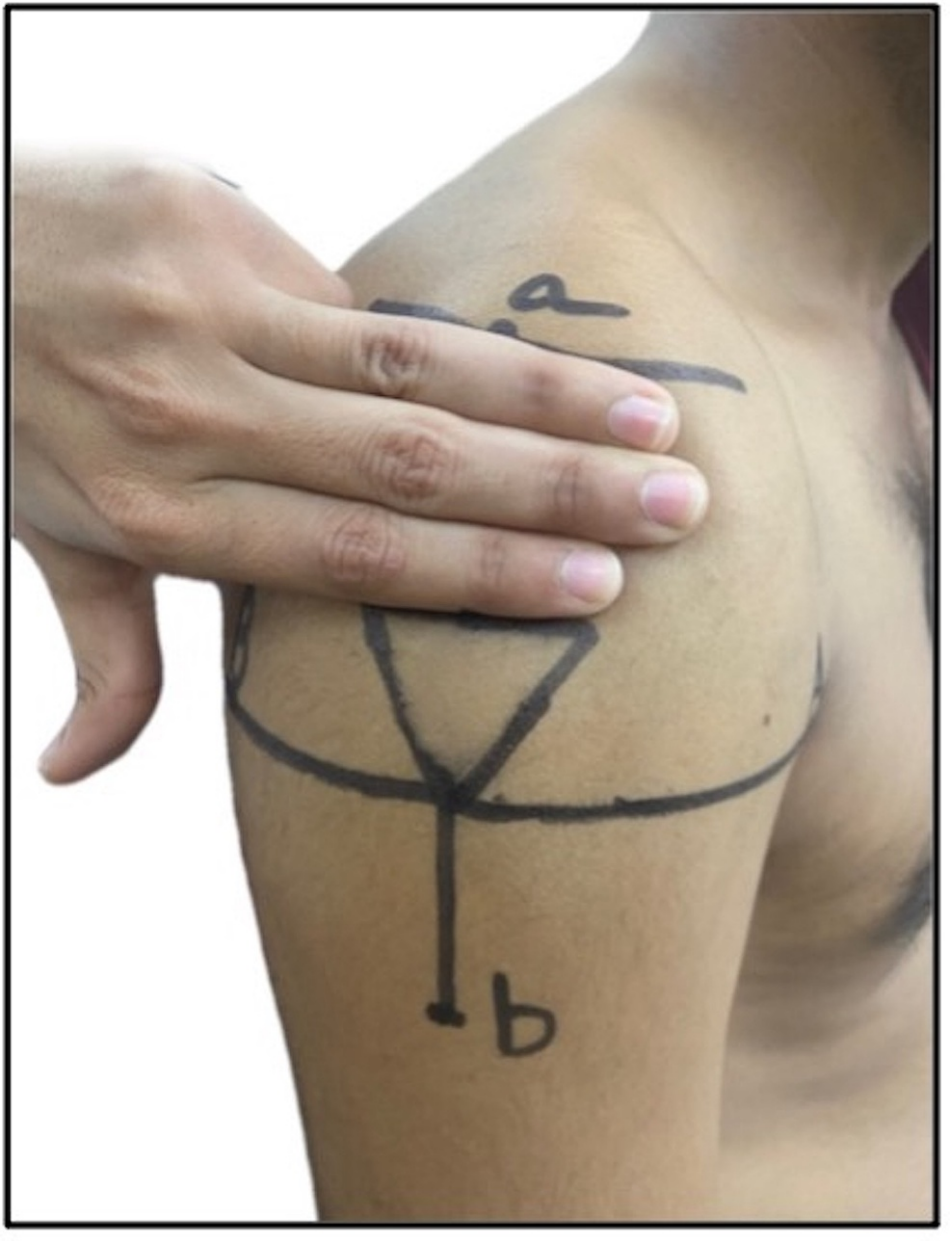
Triangular injection site, formed by an apex based on a line drawn laterally from the upper end of the anterior axillary line and base positioned on a line 3 fingerbreadths (5 cm) below the acromion: a) mid-acromion; b) deltoid tuberosity.

**Figure 5:**
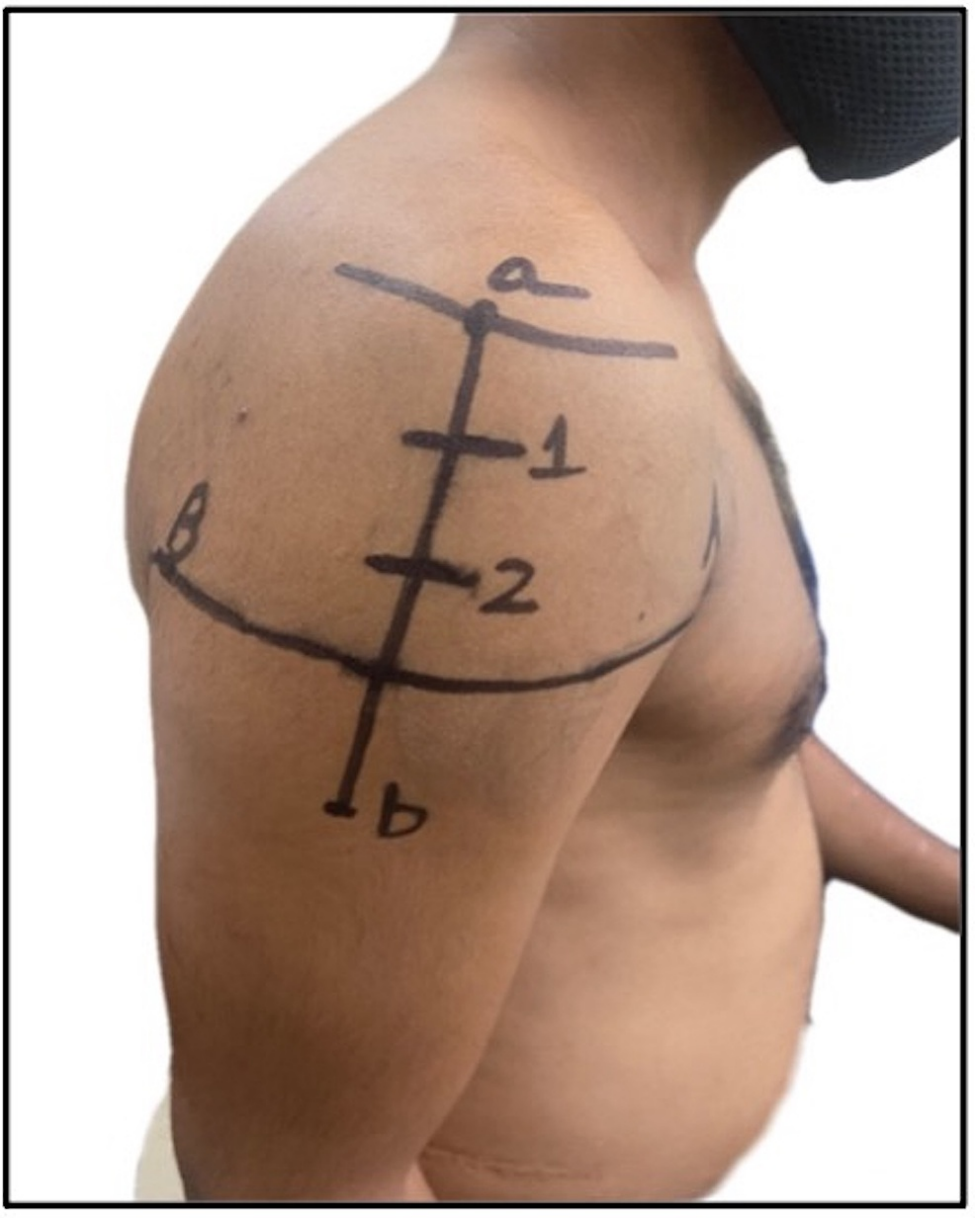
Site for intramuscular injection at the middle third of the deltoid muscle taking the acromion as the origin of the deltoid and the deltoid tuberosity as the insertion of the deltoid muscle: a-l) upper one-third; 1-2) middle one-third; and 2-3) lower one-third.

**Figure 6:**
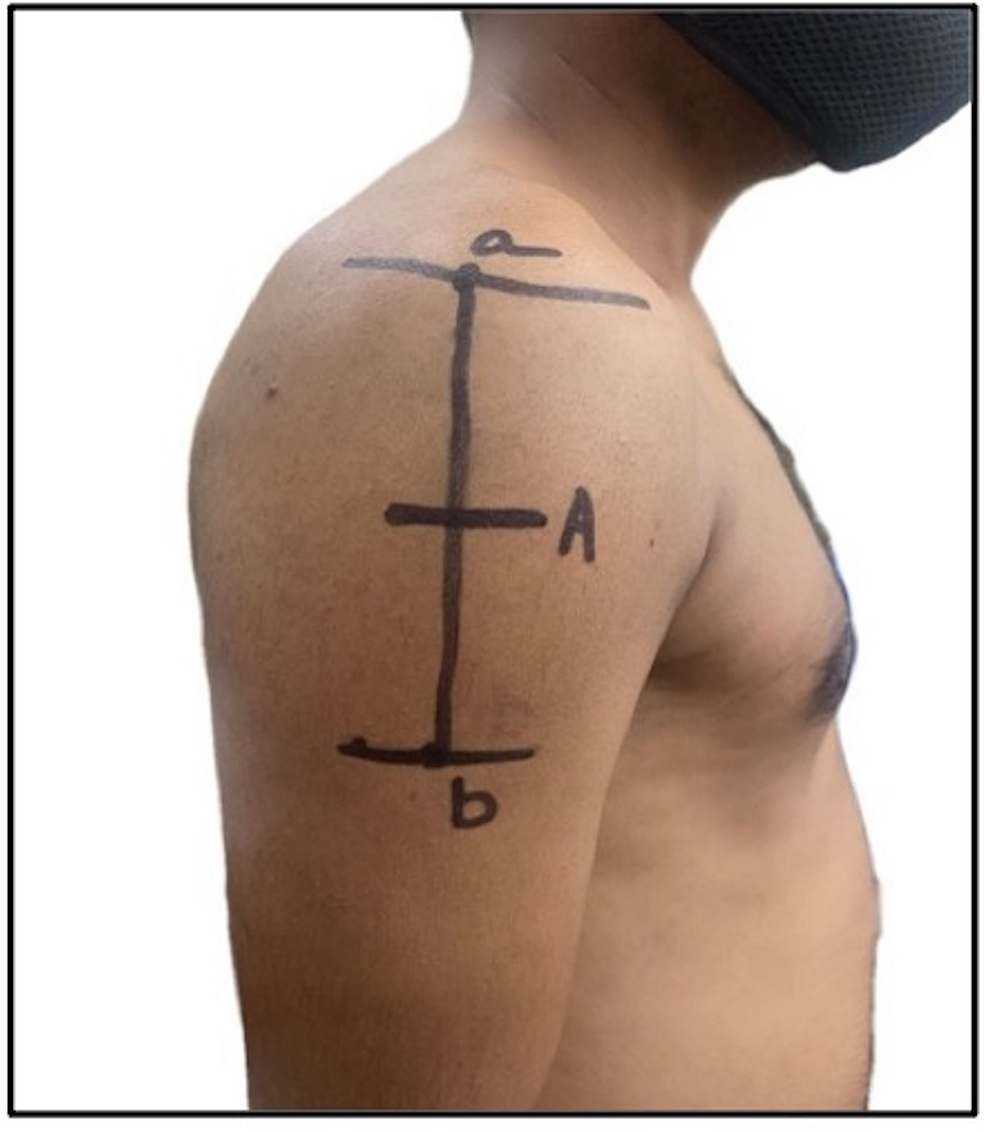
A mid-deltoid intramuscular injection site, considering acromion as the origin of the deltoid muscle and the deltoid tuberosity as its insertion: a) midpoint of lateral border of acromion; b) deltoid tuberosity; A) midpoint (5.2 cm from the acromion).

**Figure 7:**
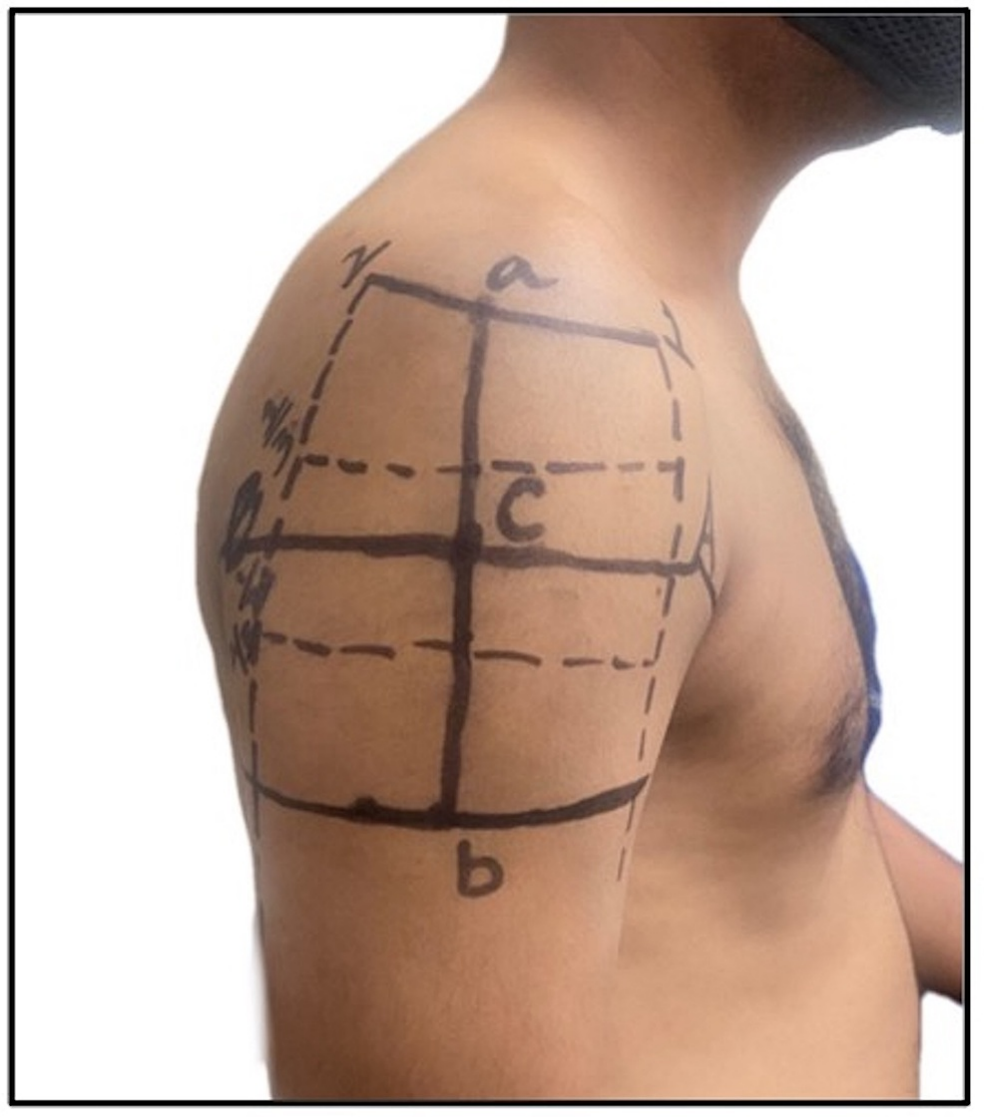
Zone between the halfway point of a-C line and the lower one-third of the a-b line may be safe for intramuscular injections.

**Figure 8:**
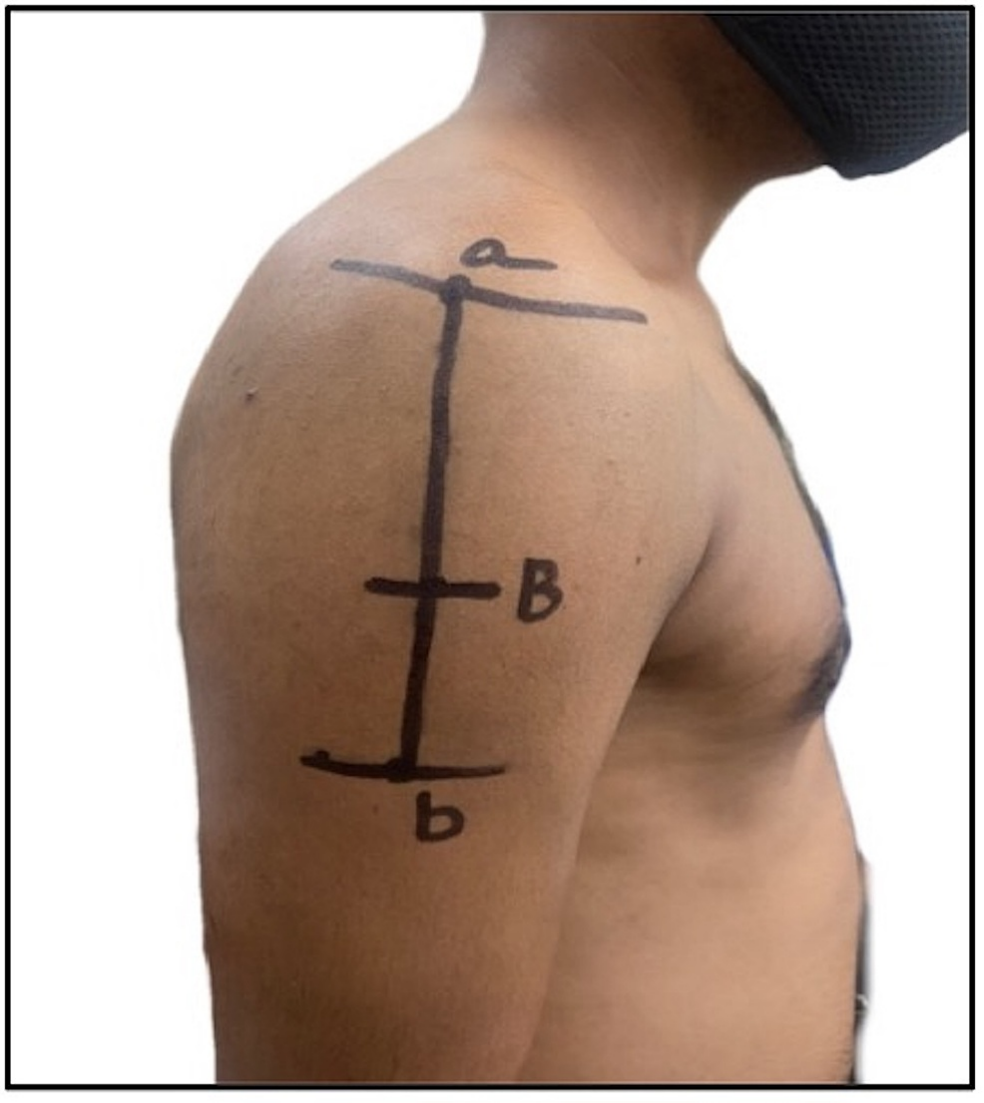
An intramuscular injection site, 7.4 cm below the mid-acromion (relatively safe much below the course of the axillary nerve and position of the subacromial/subdeltoid bursa): a) lateral border of acromion; b) deltoid tuberosity; B) 7.4 cm from the acromion border.

**Figure 9:**
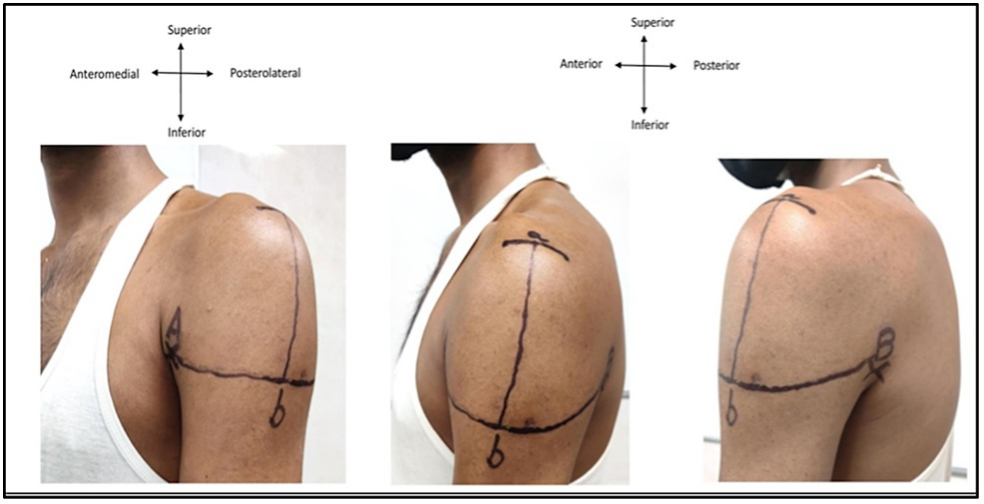
Left upper arm: A) upper end of anterior axillary line (AAL); B) upper end of posterior axillary line (PAL), a) midpoint of acromion process; b) intersection point.

**Figure 10:**
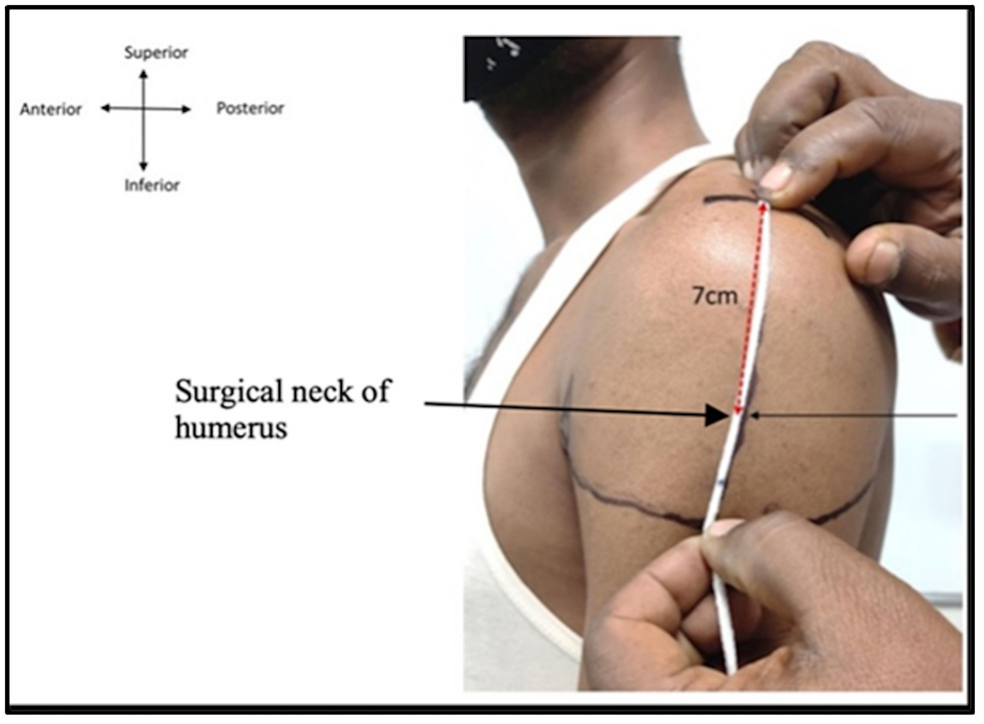
Left upper arm where the distance measured from point a to b is 11 cm (white thread) and surgical neck of humerus (red dotted line) is 7 cm from point a.

Characteristics of Included Studies

Tables 3-5 summarize the characteristics of the selected studies. Of the selected 39 articles, 18 articles focused on the administration of deltoid IMIs (Shown in Table 3). These 18 comprised two public surveys, three cadaveric studies, four case reports, four review studies, one book chapter, and four cross-sectional/cohort studies. Of the selected 39 articles, seven articles focused on the variations in the underlying neurovascular structures in proximity to the deltoid muscle (Shown in Table 4). These seven publications comprised three observational studies, one cadaveric study, two book chapters, and one immunization report. The remaining 14 publications were the immunization guides issued by the Indian and international public health organizations and the data obtained from them was used for comparison. Among the 39 reviewed articles, only 12 articles focused specifically on the site of deltoid IMI. Table 5 summarizes the data extracted from these 12 articles regarding the sites, surface landmarks, techniques, and post-injection complications. All the proposed sites in these 12 publications were demonstrated on our study volunteers who underwent surface marking of the bony, soft tissue structures. The results section describes the images that were taken.

Risk of Bias Assessment

The studies selected for this review study used a variety of methodological techniques. All three phases of the ROBIS tool were utilized to assess the risk of bias in our review study’s methodology. Except for a few points in Domain 2 of Phase 2, all other requirements were met. As a result, the total risk of bias was determined to be minimal.

Discussion

The proposed techniques and observations in all these studies were considered in the context of the guidelines recommended by several national public health agencies and regulatory bodies across India and the world. The Centers for Disease Control and Prevention (2021) and National Immunization Technical Advisory Groups (NITAGs) in Ireland (2020) and New Zealand (2020) advised that the correct site to insert the needle in the deltoid muscle is in the central and thickest portion of the muscle, which lies in the center of a triangle (Figure 4), the base of which is formed by the lower edge of the acromion process and the apex direct downwards at the crease of the axillary fold/armpit [40]. However, a review of deltoid anatomy and the AXN showed that AXN is usually 5-7 cm below the acromion tip, but the distance was between 4.34 cm and 6.39 cm, with an average distance of 5.58 cm [41]. The nurse who administers a deltoid IMI 5 cm below the acromion can therefore only be millimeters from the AXN.

According to the Canadian Immunization Guide (Modified 2020), issued by the Government of Canada, the deltoid muscle is the preferred site for IMI in adults and adolescents older than 12 years, whereas this site is not recommended for children aged 12 months and younger [42]. As per the guidelines issued by NITAGs in Australia (2018), the anatomic site recommended for deltoid IMI is a smaller triangle-shaped area in the middle of the deltoid, above the deltoid tuberosity. This site is located midway between the acromion and deltoid tuberosity, in the middle of the muscle [43]. McGarvey and Hooper (2005) stated that the subdeltoid bursa extends 5 cm below the acromion process, so the midpoint of the deltoid IMI site may be dangerous [10].

The US Department of Health and Human Sciences Centers for Disease Control and Prevention (2017) states that the midpoint of the deltoid is about 2 inches (or 2-3 fingerbreadths) below the acromion process and above the armpit in the middle of the upper arm. This area is the central and thickest portion of the deltoid muscle and is the recommended site for IMI [44]. However, this site could be dangerous due to the risk to subacromial bursa [10]. The National Health and Medical Research Council in the Australian Immunization Handbook (2015) proposed for IMI a region in the middle part of the deltoid muscle with acromion as the beginning of the deltoid muscle and tuberosity of the deltoid muscle as its insertion [45]. However, this site also could be dangerous due to the risk to subacromial bursa [10].

Based on the neurovascular network lying underneath the deltoid muscle and in relation to the upper end of the humerus, the proximal humerus is related to a network of arteries arising from the second part of the axillary artery. The PCHA, along with the AXN, is frequently found in the region between 5 and 9 cm below the lateral border of the mid-acromion process. Hence, an alternative site (site “b”) is proposed which is 5 fingerbreadths (more than 10 cm) below the midpoint of the lateral border of the acromion (Figures 9-10). This site is far below the surgical neck of the humerus (7 cm), the AXN (7 cm), the subdeltoid bursa (5 cm), and the PCHA (7.6 +/-1.0 cm); therefore, the chance of injury to blood vessels and nerves is the least. The intersection of the anteroposterior axillary line (the line connecting the upper end of the anterior axillary line and the upper end of the posterior axillary line) and the perpendicular line of the middle acromia is like the site proposed by a study in Japan [1] as the most suitable site for IMI. Further cadaveric and ultrasonographic studies are needed to study the neurovascular profile in relation to safer areas for IMI in the deltoid muscle.

Limitations

Free access to complete text was unavailable for several articles; thus, a review of such articles could not be performed because abstracts were excluded from our study. This limitation shall be overcome in our future review articles.

## Conclusions

Based on findings from our literature review for deltoid IMI sites and techniques, we conclude that the area around the shoulder joint and up to the lower level of the intertubercular sulcus is highly vascular, and the presence of many anomalous patterns of arteries in this area is not rare. We propose an alternative site (site “b”) that lies 5 fingerbreadths/10 cm below the midpoint of the lateral border of acromion as the safest site to avoid injury to the AXN, PHCA, subacromial and subdeltoid bursae, shoulder joint, and radial nerve. We believe that our proposed site for IMI can be useful for clinicians in a daily clinical practice setting.
